# Potentially Prolonged Psychological Distress from Postponed Olympic and Paralympic Games during COVID-19—Career Uncertainty in Elite Athletes

**DOI:** 10.3390/ijerph18010002

**Published:** 2020-12-22

**Authors:** Anders Håkansson, Karin Moesch, Caroline Jönsson, Göran Kenttä

**Affiliations:** 1 Depterment of Clinical Sciences Lund, Psychiatry, Faculty of Medicine, Lund University, S-22100 Lund, Sweden; 2 Region Skåne, Malmö Addiction Center, Clinical Sports and Mental Health Unit, S-20502 Malmö, Sweden; karin.moesch@rfsisu.se (K.M.); caroline.jonsson@skane.se (C.J.); 3 Swedish Sports Confederation, 114 33 Stockholm, Sweden; goran.kentta@gih.se; 4 Department of Psychology, Faculty of Social Sciences, Lund University, S-22100 Lund, Sweden; 5 FIFPRO (Global Representative for Professional Football Players), 2132 Hoofddorp, The Netherlands; 6 Spelarföreningen (National Representative for Football Players), 433 63 Sävedalen, Sweden; 7 The Swedish School of Sport and Health Sciences, 114 33 Stockholm, Sweden; 8 School of Human Kinetics, University of Ottawa, Ottawa, ON K1N 6N5, Canada

**Keywords:** COVID-19, elite athlete, Olympic games, Paralympic games, psychological distress, sports psychology, mental health

## Abstract

The coronavirus disease 2019 (COVID-19) pandemic has had a significant impact on the world of sports due to periods of home quarantine, bans against public gatherings, travel restrictions, and a large number of postponed or canceled major sporting events. The literature hitherto is sparse, but early indications display signs of psychological impact on elite athletes due to the pandemic. However, beyond acute effects from lockdown and short-term interrupted athletic seasons, the postponed and still uncertain Olympic and Paralympic Games may represent a major career insecurity to many athletes world-wide, and may lead to severe changes to everyday lives and potentially prolonged psychological distress. Given the long-term perspective of these changes, researchers and stakeholders should address mental health and long-term job insecurity in athletes, including a specific focus on those with small financial margins, such as many female athletes, parasports athletes, athletes in smaller sports, and athletes from developing countries. Implications and the need for research are discussed.

## 1. Psychological Consequences of COVID-19 in Athletes 

The spread of the SARS-CoV-2 virus, and the morbidity and mortality related to the coronavirus disease 2019 (COVID-19), constitutes an unprecedented threat to public health, financial systems, and occupational conditions world-wide. Beyond the obvious consequences of the virus and the severe physical disease surrounding it, researchers have expressed a growing awareness about COVID-19 as a threat against mental health in the population [1]. 

While the COVID-19 crisis affects many (or virtually all) financial sectors in society, specific professions are more affected by the COVID-19 pandemic than others, particularly through feelings of insecurity about future working conditions [2]. The world of competitive sports at the highest level—including a wide range of professional, semiprofessional, and highly engaged athletes—is one of these occupational sectors clearly affected by the pandemic. Although they perform on the highest international level, the financial security, conditions, and possibilities for a professional career are highly diverse across the globe, and across sports and genders. Moreover, job insecurity is likely to substantially affect mental health in athletes and coaches [3]. The present paper aims to raise topics related to the prolonged psychological consequences for elite athletes and aspiring elite athletes, and for broader sectors involving many professionals around them, related to the previously unseen and prolonged uncertainty regarding the Olympic and Paralympic Games due to the COVID-19 pandemic. A number of studies focused on early responses to the pandemic in the context of sports. However, the effects of the currently prolonged COVID-19 pandemic, including a current surge in virus spread, potentially leading to prolonged uncertainty, professional insecurity and distress, is unknown. The current pandemic causes an unprecedented situation of career insecurity in athletes. Moreover, it also presents a challenge for engagement in sports, physical exercise, and well-being at all levels. Large sporting events like the Olympic and Paralympic Games have been suggested to stimulate physical activity and sports participation in the general population. While some government reporting has supported this idea—exemplified by an increased sports focus in the population around the 2012 London Olympics [4]—the research in this domain is still insufficient and inconclusive. Despite limitations in research design, some improvement in attitudes toward physical activity has been reported around such events [5]. Researchers have pointed out physical activity fueled by the Olympic spirit as an overarching health priority in general [6], and specifically in COVID-19 times [7]. 

An emerging line of scientific literature has highlighted the profound impact of the pandemic, particularly on the macrolevel business situation in the world of sports [8]. Above that, psychological consequences may arise from physical restrictions like stay-at-home orders during phases of lockdown in many countries, from becoming socially isolated in other countries due to travel bans, and from the postponement, cancelation, or marked alteration of many sporting events due to COVID-19-related bans against gatherings of sports audiences. From a historical perspective, the COVID-19 pandemic represents one of the most extensive changes to ever occur in the world of sports [9]. 

Researchers have documented early social media experiences of many top athletes supporting the need for action against the pandemic in a tone of accepting the current situation and engaging in what is important in society outside of sports [10]. However, prolonged impact from the pandemic is likely to increase the pandemic’s negative impact on sports at all levels. Clearly, the shortfall of income from selling tickets to live sporting events, and the high level of uncertainty about the conditions of future events, may have a severe financial impact on the wider domain of sports and society, affecting elite athletes as well as children and adults who participate in recreational sporting activity. In addition, the COVID-19 crisis in some cases has put additional pressure on athletes because of the need to accelerate league seasons, leaving less time for physical and mental recovery between games, which could potentially contribute to an increased risk of sports-related injury and fatigue. In addition, sports medicine experts have raised the concern in media that a more severe course of COVID-19 may cause an athlete to end their professional career in endurance sports. During the early stages of the pandemic, in addition to a broad range of COVID-19-related advice and guidelines published within the domain of sports by different stakeholders internationally, concerns about physical and psychological health consequences in athletes were also raised within the scientific community [11,12]. Early guidelines for athletes during the pandemic have tended to predominantly address physical needs [13], although suggested guidelines for mental health have also been published [14]. Altogether, these publications tend to highlight the consequences associated with the acute phases of lockdown and home confinement, the obvious limitations to sports performance and exercise during those times [15], physical consequences and risks of COVID-19 transmission during sports [16], physical aspects of lockdown and return to sports after these restrictions [17,18], or the challenge of traveling and mass gatherings in times of the pandemic [19]. Thus, further attention needs to address the psychological impact on athletes, especially the prolonged effects beyond the acute phases of the pandemic. Specifically, guidelines for the assessment and treatment of mental health symptoms in athletes during COVID-19 have been published [14]. 

Prior to the particular situation of COVID-19, psychological distress and mental health in athletes have received increasing attention from researchers and relevant stakeholders in recent years. While the actual prevalence of mental health disorders in athletes is considered to be at a similar or slightly higher level than in the general population, its clinical picture may be different: stigmatization may be higher, help-seeking lower, and opportunities for professional and specialized treatment may be more limited. This has led to the overall interest in better assessment and treatment of poor mental health in athletes and in establishing specific chains of care for this particular group [20,21,22,23]. While there is increasing knowledge about sport-specific and personal risk and protective factors for athletes’ mental health [24], no research has addressed the consequences of a prolonged period of career uncertainty, the postponement and cancellation of major events, and an ongoing pandemic situation.

The scientific literature is hitherto limited with respect to actual psychological effects on athletes from the lockdown in sports. However, in an online survey carried out in May–June 2020, a high percentage of top league players from three major team sports in Sweden reported a self-perceived negative psychological impact from the COVID-19 situation. A minority reported having increased their alcohol consumption and gambling during the pandemic, although no data are available for comparison. Importantly, more clinical measures of depression and anxiety were associated with self-reported worry about one’s own future in sports in the context of the COVID-19 pandemic. While the conclusions were limited by a low response rate, the study provided indications of negative psychological influence in three team sports highly affected by the acute phase of the lockdown situation [25]. A multicenter European survey study in soccer players, hitherto only reported by the media, showed elevated rates of depressed mood and anxiety [26]. Likewise, studies in handball players documented that psychological factors may affect exercise and recovery during the COVID-19 period [27,28]. In one study that was limited by the use of brief screening questions, a substantial number of South African athletes reported depressed mood and loss of energy during lockdown [29]. Findings from a study in Italian athletes showed a significant increase in athletes’ perception of stress at the onset of the pandemic [30].

## 2. Potential for Prolonged Distress and Extended Uncertainty in Olympians and Paralympians

Long-term consequences beyond relatively brief periods of confinement and the acute phase of the pandemic may persist and develop further. Among the major structural changes occurring in the world of sports was the cancelation and discontinuation of major tournaments and full seasons or playoffs in team sports world-wide. One of the relatively early consequences was the decision to postpone the 2020 Summer Olympics and Paralympics in Tokyo [31]—a decision likely seen as unsurprising given the travel bans and widespread limitations on public gatherings. Fear of physical injuries as a consequence of the pandemic in Olympians has been expressed [13,32]. With few exceptions [33], little research has addressed the potential mental health consequences from the postponement of these major events. Before the outbreak of the pandemic, aspects of mental health in Olympic and Paralympic athletes in general have been discussed in light of the different stages in the four-year quadrennium between these events [34]. Dividing the quadrennium into the stages before, during, and after the Olympics/Paralympics emphasizes how important the given time structure is for the Olympic/Paralympic mind. With COVID-19, this time structure collapsed, and the 2021 competition year still remains highly uncertain.

Since the decision to postpone the Games, the impact of COVID-19 on the lives and working conditions of Olympic and Paralympic athletes continues because of severe restrictions and challenges, with the pandemic being prolonged and unlikely to diminish in the near future. The number of cases globally, and on more than one continent, displayed a new surge during autumn 2020 [35], leaving athletes uncertain of how to plan for the Olympic and Paralympic games currently scheduled for summer 2021. Given the remaining uncertainty about the 2020/2021 competitive season and larger events, this is also likely to have an impact on the preparations for the 2022 Winter Olympics and Paralympics [36]. It can be argued that the overall challenge of the global pandemic is likely to create uncertainty about similar large-scale events in the future. Thus, in comparison to many other occupational consequences of the pandemic, the impact on the everyday lives of athletes and their expectations for the future is likely to be extended and subject to possible uncertainty for a long time.

As the planning for the 2020 Tokyo Olympic and Paralympic Games can be re-activated in the event of a more favorable pandemic situation, such a decision will have to take into account the highly diverse conditions for qualification and preparation world-wide, based on diverse phases of the COVID-19 spread. In addition, to reiterate, the insecurity about when and how such an event will be carried out may still be prolonged and may again be subject to further insecurity in the case of repeated surges in the pandemic. There is reason to believe that such feelings of uncertainty may lead to increased psychological distress. Handling insecurity is challenging for most people, and based on current cognitive models in generalized anxiety, an intolerance for insecurity can lead to an increase in worrying [37,38]. In addition, financial challenges to the world of sports have been substantial in many settings, potentially affecting athletes’ psychological health even if the Olympic and Paralympic Games are to occur.

The profound impact of the pandemic on the Olympic and Paralympic Games can be considered to represent a particular threat to athletes’ mental well-being, due to the nature of these events as a long-term and fundamental motivational target for athletes. Few professional areas affected by the COVID-19 pandemic are characterized by something similar to this four-year preparation cycle, now suddenly disrupted by COVID-19-related restrictions and subject to a high degree of job insecurity. Previous experience from the Olympic Games shows the nature of the Games as a key component in athletes’ careers [39,40], where career plans for many athletes are measured in these four-year periods, making these major sports events particularly sensitive as a motivational factor, which disappeared suddenly during the pandemic. Researchers in the area have suggested that retiring from an elite athletic career may be one of the likely options for athletes affected by the postponed Games [41]—a scenario even more realistic in the case of a prolonged and uncertain postponement of these and other large events. In many cases, a sports career represents the financial security of the individual and their loved ones; thus, the socioeconomic situation of concerned significant others may also be at stake when sporting careers are put on hold for an uncertain period of time.

Given the early research data on psychological distress in athletes due to the COVID-19 pandemic [25,26,30], this crisis is likely to have prolonged and accumulative effects on Olympic and Paralympic athletes in the sense that worry about their own sport, and about their career in sports, is likely to occur. Moreover, dual career planning (i.e., the planning for an academic or professional career outside of or after the active sports career) is already known to be a challenge for many athletes, and the overall financial crisis related to COVID-19 is likely to make job opportunities even more difficult to come across in the situation of transitioning from sports to other careers. Dual careers have been shown to be of significant importance to athletes during and after their career, but they may be a challenge, potentially with larger difficulties in female athletes than in their male counterparts [42].

In a study in team sports players in Sweden, a majority of respondents confirmed feeling concerned about their sport and about their future in sports due to the pandemic [25]. However, little is known about how the impact on sports events, including the Olympic and Paralympic Games, affects athletes in individual sports, or athletes coming from developing countries. Moreover, in sports settings with limited resources, sport psychological support may be less accessible and less structured. Overall, little research has highlighted the mental health support in low-income countries [43,44], and the prevention and treatment of sports-specific mental health needs in such settings are likely to present a challenge compared to high-income settings. Thus, athletes in developing countries may be more vulnerable to the shortfall of career targets such as postponed or canceled major sporting events. Beyond sports, the COVID-19 pandemic itself may cause particular challenges in developing countries, where, for example, vaccine distribution may be a particularly large challenge [45]. Therefore, negative long-term effects on sports may be substantial.

In addition, concerns have been raised about women’s sports being more strongly affected than men’s sports. The markedly increased challenge in women’s sports is based on the large gap in sponsorship income and other financial resources, as well as more challenging professional career opportunities for women and the lower worldwide media coverage of women’s sports than men’s sports. Altogether, these differences, evident prior to the current crisis, are likely to leave female athletes with narrower margins in the COVID-19 crisis [46]. These concerns, although hitherto rarely researched, seem to be consistent with the findings of a recent survey in Swedish top-league athletes where women were more likely than men to express worry about their future in sports and markedly more likely to screen positive for depressive and anxiety symptoms [25].

From a sporting event organizer’s standpoint, the COVID-19 pandemic is likely to affect many business sectors surrounding the travel and tourism industry, especially around large sporting events [47]. More specifically, groups that are financially dependent on the tourism industry may suffer [48]. Thus, job insecurity, financial loss, and mental distress are likely to affect various populations and professions beyond those involved in the domain of competitive sports itself.

## 3. Directions for Research, Prevention, and Mental Health Treatment

As the pandemic continues to spread globally, further enhanced by the surge of cases during late autumn and winter 2020, policy makers, clinical workers, and researchers have reason to actively consider the mental and occupational health of professional and semiprofessional athletes. It is becoming increasingly clear that such mental health concerns will have to be assessed far beyond the acute effects of regional or national lockdown decisions, or of home confinement during early COVID-19 phases. Importantly, psychological distress from COVID-19 in sports may need to be studied in the context of job and career insecurity, and in the context of a prolonged career-limiting situation of an uncertain duration and an unknown outcome. Beyond the career, an involuntary and unpredictable end of the career—potentially brought on by the COVID-19-related impact on sports—may be associated with difficulties in the transition from sports to another career, and this merits further research. Likewise, decisions to plan for a family may depend on the outcome of specific sporting events, where the disruption of the Olympic quadrennium may have a profound impact on such private-life decisions. When career termination is involuntary, as can happen during the pandemic situation and the cancellation of major sports events, it is considered to lead to a more problematic transition process [49]. Therefore, active follow up of dual-career opportunities will be needed.

Psychological research on the impact of COVID-19 on athletes may need to focus on the long-standing consequences of the postponed and possibly canceled Olympic and Paralympic Games. Researchers have previously underlined the need to keep up therapeutic contacts with elite athletes in need of support throughout the pandemic [14]. While counseling services may already be familiar with digital solutions in the context of sports psychology—typically due to the high degree of mobility in this group—such methods may still present certain challenges to the structural assessment and therapeutic work in athletes psychologically affected by the COVID-19 crisis. Thus, beyond research initiatives assessing the impact on psychological health, such preventive and therapeutic initiatives are important to develop during and after the current crisis. Recent years have seen emerging attention paid to supporting the mental health of athletes. Strategies suggested for prevention—amongst other regular mental health screenings—include increasing mental health literacy in all individuals around athletes, educating athletes about topics like stress management, promoting psychological skills training, recovery management (e.g., sleep hygiene), and strategies allowing athletes to cope with insecurity. Strategies for treatment include the establishment of sport-specific support systems providing tailored treatment for elite athletes that are both firmly established in the elite sport context and easily accessible [14,21].

From a COVID-19 perspective, such strategies need to be intensified and should further specifically address the peculiar and context-specific challenges that athletes face in the current pandemic—that is, the insecurity regarding the Olympic and Paralympic Games and other upcoming major sporting events—and the effect of that insecurity on the individual’s life and career planning. Moreover, a special focus should be placed on the development and worsening of more severe clinical psychiatric manifestations due to the current situation. Therefore, relevant stakeholders are the domain of sport psychological support both in the applied setting and in clinical treatment units.

In addition to a response to the effects of uncertainty while waiting for a decision, interventions may be needed if the Olympic and Paralympic Games are ultimately canceled. This would represent a previously unseen scenario (except for times of World War [33]), and a potential cancelation may distinctly evoke strong emotional reactions and changes to everyday lives and careers of many athletes, coaches, and other professionals preparing for these major sporting events. Even without such a decision, a crisis intervention plan may need to be prepared for sports psychology facilities and for other stakeholders within the world of sports. Thus, given the difficulty in establishing dual careers, an action plan in the currently prolonged COVID-19 situation may need to take into account athletes’ potential need for dual-career planning, financial support in case of a shortfall of incomes, and mental health support in case of a sudden and distinct career change. For specific settings and for sports sectors with limited financial resources, such a crisis plan may be needed even if the Games are not fully canceled but instead extensively restructured or limited in volume. Thus, such an action plan in the area may also need to consider the shortfall of sponsoring opportunities as well as reduced ticket vending for sports events, and the major challenge this presents to the personal financial situation of athletes and their families. Such active dual-career-facilitating interventions may specifically need to target female athletes and athletes in financially deprived settings.

Moreover, as pointed out previously, in times of a crisis like the COVID-19 pandemic, business management surrounding the world of sports may need to be innovative in order to lessen the harm caused by the pandemic [8]. Thus, beyond the personal consequences of athletes, policy making and research may need to assess entrepreneurs in many businesses related to and embedded in large sporting events, as the impact on the travel and tourism industry surrounding such events is so large [47,48]. Thus, an action plan preparing for either postponed or canceled Games should take a number of stakeholders outside the inner circle of athletes and coaches into account.

In brief, recommendations for stakeholders within and surrounding the world of sports may be (1) to provide career support for athletes who are affected by the postponement; (2) to increase sports psychology support to meet the needs of the athletes, with a special focus on coping with insecurity about their career and their future; (3) to provide access to mental health providers in specialized or general psychiatric facilities for athletes who suffer from clinical symptoms; and (4) to formulate regional and global action plans for the potential impact on occupational situations and health in athletes and other affected stakeholders in the case of a final cancelation of the Olympic and Paralympic Games. In addition, a gendered approach to all stages of prevention and intervention in athletes’ mental health is of great value.

## 4. Conclusions

Beyond the likely psychological effects on elite athletes from early phases of confinement and canceled sporting events during the COVID-19 crisis, the prolonged and highly uncertain future of the Olympic and Paralympic Games presents a potentially large mental challenge to athletes. Emerging data suggest that psychological distress in athletes is one of the consequences of the pandemic, and more knowledge is needed both in athletes in general, and with particular focus on the highly specific situation for athletes striving for the Olympics and Paralympics. Research, prevention efforts, and treatment interventions may need to focus on this uncertainty in the context of job insecurity, as athletes represent one of the occupational sectors most clearly affected. The everyday lives of many athletes—typically strictly planned and often involving a focus on the fixed time frame surrounding the typical Olympic quadrennium—are likely to be severely altered by this insecure situation. It is also important that research, prevention, and treatment initiatives involve gender aspects.
